# Rapid and visual detection of milk vetch dwarf virus using recombinase polymerase amplification combined with lateral flow strips

**DOI:** 10.1186/s12985-020-01371-5

**Published:** 2020-07-11

**Authors:** Yuhao Cao, Dankan Yan, Xinyang Wu, Ziqiang Chen, Yuchao Lai, Lanqing Lv, Fei Yan, Jianping Chen, Hongying Zheng, Xuemei Song

**Affiliations:** 1 State Key Laboratory for Managing Biotic and Chemical Threats to the Quality and Safety of Agro-products, Key Laboratory of Biotechnology in Plant Protection of Ministry of Agriculture and Zhejiang Province, Institute of Plant Virology, Ningbo University, Ningbo, 315211 China; 2 College of Animal Science and Technology, Nanjing Agricultural University, Nanjing, 210095 China; 3 Department of Biochemistry and Molecular Biology, Zhejiang Key Laboratory of Pathophysiology, Medical School of Ningbo University, Ningbo, 315211 China

**Keywords:** Milk vetch dwarf virus, Polymerase chain reaction, Recombinase polymerase amplification, Lateral flow strips, Rapid and visual detection

## Abstract

**Background:**

Milk vetch dwarf virus (MDV) is an important ssDNA virus which causes yellowing, stunting and leaf rolling symptoms on legumes. In China, the virus causes great economic losses and has recently been found to infect tobacco. The expansion of its host range and its ability to spread rapidly has given rise to the urgent need for a sensitive, specific and rapid diagnostic assay that can assist in effective disease control.

**Methods:**

Assays based on the polymerase chain reaction combined with lateral flow strip detection (PCR-LFS) and recombinase polymerase amplification combined with LFS (RPA-LFS) were developed targeting the coat protein (CP) gene of MDV.

**Results:**

The PCR and RPA assays could detect respectively 10^3^ copies or 10^1^ copies of MDV by agarose gel electrophoresis. The PCR-LFS and RPA-LFS assays developed could both detect as few as 10^1^ copies per reaction at 37 °C. Both methods could detect MDV in crude leaf extracts.

**Conclusions:**

The RPA-LFS assay developed is a rapid, sensitive and specific method for detecting MDV, which is convenient and has great potential for use in the field.

## Background

Milk vetch dwarf virus (MDV) is a nanovirus that causes yellowing, stunting and leaf rolling symptoms on legumes. Its genome consists of eight circular single-stranded DNAs, each about 1 kb in length and containing a single potential open reading frame (ORF). All DNA components have a 29–34 base region capable of forming a stem-loop structure that has been consistently identified in the genetic components of nanoviruses, geminiviruses and in porcine circovirus (PCV) [1]. MDV was first reported from *Astragalus sinicus* in Japan in 1968 [2]. In China, serological tests first demonstrated in 2007 the presence of a nanovirus in faba bean (*Vicia faba* L*.*) growing in Yunnan Province and which was showing leaf yellowing and rolling and plant stunting [3] and in 2010 further molecular tests confirmed the presence of MDV [4]. During 2014–2017, MDV was also found infecting cowpea (*V. unguiculata* L. Walp) and broad bean (*V. faba* L.) plants in Anhui, Zhejiang and Shanghai [5]. Surprisingly, in Shandong and Gansu province during 2016–2017, MDV was also detected in field-grown tobacco (*Nicotiana tabacum*) showing virus-like symptoms including dwarfing and leaf wrinkling [6, 7], the first record of the virus from a non-leguminous host. Large economic losses caused by this virus, its gradual geographical spread and the expansion of its host range aroused our concern, and we therefore wanted to establish a method for rapid detection of MDV as soon as possible to facilitate surveillance of the virus.

A variety of molecular techniques have been used to detect MDV genomic DNA in plant tissues, including the polymerase chain reaction (PCR) and rolling circle amplification (RCA) [1, 4, 7]. However, these methods are either time consuming or require complex and expensive laboratory instruments, which make them difficult to apply under field conditions. Recently, a method was reported for the rapid detection of MDV using loop-mediated isothermal amplification (LAMP) combined with the addition of SYBR Green I to the reaction product [8]. This provides a possible detection method for the field diagnosis of MDV using a portable heating apparatus.

Recombinase polymerase amplification (RPA) is a recently-developed nucleic acid amplification technology [9]. This method provides highly specific DNA amplification of very small amounts of target DNA, to reach detectable levels in minutes at a constant low temperature (37–42 °C) [10, 11]. RPA products can then be detected by agarose gel electrophoresis [9], and can be quantified using TwistAmp™ exo probes (TwistDx, Cambridge, UK) [12–14] or simply with a lateral flow dipstick assay (MileniaBiotec, Giessen, Germany). Since the amplification product can be visualized by a lateral flow strip (LFS) if a specific probe is added into the RPA reaction solution, RPA combined with LFS (RPA-LFS) stands out as a convenient technique that is suitable for use in the field [15].

In this study, we established an RPA-LFS assay targeting the coat protein (CP) gene for the detection of MDV DNA-S. We then evaluated the specificity and sensitivity of this assay compared with that of conventional PCR combined with LFS (PCR-LFS). This is the first report showing that an RPA-LFS assay can be used to detect MDV.

## Methods

### Sources of viral samples

Cowpea plants with stunting and leaf-rolling symptoms were collected from Hefei city, Anhui province in 2017 and 2020, and samples were frozen in liquid nitrogen and stored at − 70 °C. *N. benthamiana* plants infected with tomato yellow leaf curl virus (TYLCV) were kindly provided by Dr. Xueping Zhou. *N. benthamiana* plants infected by rice stripe virus (RSV) were planted in our greenhouse. Some virus vectors such as turnip mosaic virus (TuMV) full length cDNA infectious clone vector: p35Tunos, tobacco mosaic virus (TMV) full length cDNA infectious clone vector: p35S-30B::GFP and porcine circovirus (PCV) full length cDNA clone vector: pEASY-T5-PCV3 were kindly provided by Dr. Fernando Ponz, Prof. Rongxiang Fang and Xiufang Yuan, respectively. Pepper mild mosaic virus (PMMoV) full length cDNA infectious clone vector: pCB-PMMoV plasmid, cucumber green mottle mosaic virus (CGMMV) full length cDNA infectious clone vector: pCB-CGMMV plasmid were construct by ourselves and kept in our laboratory. The virus samples and corresponding viral DNA or cDNA used in this study were all stored in our laboratory.

### Sample preparation

Viral DNA was extracted from leaves of cowpea infected with MDV or *N. benthamiana* infected with TYLCV using an E.Z.N.A.® Plant DNA Kit (Omega Bio-tek, Inc. Norcross, GA).

The total RNA of *N. benthamiana* plants infected with RSV was extracted using TRIzol reagent (Thermo Fisher Scientific, US) according to the manufacturer’s protocol and the corresponding cDNA was synthesized from 1 μg of RNA using an oligo (dT) primer (21-nucleotide plus two anchoring nucleotides) and moloney murine leukemia virus reverse transcriptase (Takara Bio Inc., JP).

For the sensitivity detection, plasmid pEASY-T5-MDV which contains a 407 bp target segment in the pEASY™-T5 Zero Cloning Vector was constructed and sequenced. The purified positive control pEASY-T5-MDV plasmid (4362 bp) was used as the initial template with a DNA concentration of 31.7 ng/μL. The initial copy number was about 6.7× 10^9^ copies/μL. The plasmids were then diluted with crude leaf extracts of healthy cowpea to provide templates of 10^6^, 10^5^, 10^4^, 10^3^, 10^2^, 10^1^, 10^0^ copies/μL. The number of copies was estimated by mass concentration measured using a spectrophotometer with Avogadro constant NA = 6.022 × 10^23^ copies/mol, assuming that the molecular weight of 1 bp dsDNA is approximately 650 g/mol:

$$ \mathrm{number}\ \mathrm{of}\ \mathrm{copies}=\left(\mathrm{amount}\times 6.022\times {10}^{23}\right)/\left(\mathrm{length}\times 1\times {10}^9\times 650\right). $$

### Crude sample extraction

MDV-infected cowpea plants together with healthy cowpea plants for controls were collected from Hefei city, Anhui province in 2017 and 2020. Approximately 200 mg fresh cowpea leaf tissue of each sample was homogenized in a mesh bag (Agdia, US) with 2 mL plant lysis buffer (Tiosbio, China). The homogenized crude extract was transferred from the mesh bag into a 2 mL Eppendorf tube and briefly centrifuged. 10 μL supernatant was then transferred and mixed with 90 μL ddH_2_O, and a dilution series was prepared by sequential transfers. 1 μL supernatant of the homogenized crude extracts or their corresponding dilutions (1:10, 1:100, 1:1000, 1:10000) were used as templates. Aliquots of crude extracts were either tested immediately or stored at − 20 °C until used.

### Primers and probe design and optimizing for PCR and RPA

A pair of specific MDV primers suitable for both PCR and RPA were designed using DNAMAN Version 8 (CP1F: 5′-GTGAAGCGAATCTGACGGAA-3′ and CP1R: 5′- CATAACCTTCTTCATCTTATA − 3′) from the sequence of the MDV CP gene (GenBank accession: KY070245). These yield an amplicon size of 407 base pairs (bp). For PCR-LFS and RPA-LFS, the primers were first labeled with biotin or fluorescein isothiocyanate (FITC) at their 5′-ends, generating the forward primer CP1F-biotin and the reverse primer CP1R-FITC.

### Real-time qPCR assay

Real-time qPCR was performed in an BIORADCFX96 apparatus (BIORAD, USA) in a 20 μL reaction containing 10 μL KOD SYBR qPCR Mix, 80pM of each primer (CP1F and CP1R) and 0.8 μL DNA solution of the template. To determine the sensitivity of qPCR detection, 0.8 μL of the pEASY-T5-MDV plasmid template and its series of diluents were used, and for evaluating RPA with qPCR in field detection, 0.8 μL crude leaf extracts of each sample were used. The reaction conditions were: Pre-denaturation at 98 °C for 2 min, then 40 cycles each of 98 °C for 10s, 50 °C for 30s and 68 °C for 30s, and finally 95 °C for 15 s, 60 °C for 1 min and 99 °C for 15 s.

### MDV PCR- LFS assay

PCR was carried out in a 50 μL reaction containing 25 μL 2 × PCR buffer for KOD FX, 0.4 mM of each dNTP, 80pM of each primer, 2 μL DNA solution of pEASY-T5-MDV and 1 μL KOD FX. After the PCR reaction, 25 μL of the PCR amplification product was transferred to a new Eppendorf tube for duplicate testing, and the latex microsphere test strips (Tiosbio, China) were then directly inserted into the reaction tube. Lateral chromatography was performed for 2–4 min and the results were observed and recorded within 10 min. Positive detection of MDV was indicated by the presence of a color test line; a separate control line confirmed that the system was working properly. The ability of the LFS assay to detect MDV-PCR products from a dilution series (10^6^, 10^5^, 10^4^, 10^3^, 10^2^, 10^1^, 10^0^ copies/μL) was also explored. Reactions with crude leaf extracts of healthy cowpea were included as negative controls.

### MDV RPA- LFS assay and comparison of different incubation methods

RPA was carried out using a Twist Amp™ Basic kit (Twist Amp, Cambridge, UK) in accordance with the manufacturer’s protocols. 1 μL DNA solution of pEASY-T5-MDV plasmid was used as template and added into a 23.75μLreaction mixture containing 14.75 μL rehydration buffer, 14 mM MgAc_2_ and 0.5 μM of each primer. Reactions with crude leaf extracts of healthy cowpea were included as negative controls. The tubes were then incubated at 37 °C for 30 min in a PCR instrument, water bath or oven. The sensitivity of detection was also tested using a dilution series as before. After the reactions were completed, 1 μL of each amplification product was diluted 4000 times and latex microsphere test strips were then inserted into 40 μL of the diluted sample before incubation and examination as described above.

### Determination of specificity and sensitivity

The specificity of the primer pair was tested using the plasmid pEASY-T5-MDV and various non-target DNAs or cDNA of viruses. The templates used were: DNA of *N. benthamiana* plants infected by TYLCV, cDNA of *N. benthamiana* plants infected by RSV and the control virus plasmids including pEASY-T5-PCV3, pCB-PMMoV, pCB-CGMMV, pGR-TuMV-GFP and p35S-30B::GFP. Among these viruses, PCV is a virus with a somewhat similar structure to MDV [1, 16], and TYLCV is a geminivirus which, like MDV, can also infect tobacco [17]. TuMV, PMMoV and TMV can all infect tobacco, while CGMMV and RSV are viruses unrelated to MDV.

To explore the sensitivity of the primer pair, pEASY-T5-MDV plasmid DNA (4362 bp) with the concentration of 31.7 ng/μL was used as the initial plasmid, and ten-fold serial dilutions of pEASY-T5-MDV plasmid DNA (10^6^, 10^5^, 10^4^, 10^3^, 10^2^, 10^1^, 10^0^ copies/μL) with crude leaf extracts of healthy cowpea, that was also as a negative control, were used as templates for both PCR and RPA. Three technical repeats were made.

After the PCR reaction, 5 μL of the amplicon of each PCR product was used for agarose gel electrophoresis. After the RPA reaction, 25 μL of the amplicon of each product was mixed with 100 μL tris saturated phenol and centrifuged before using 5 μL of the purified RPA for agarose gel electrophoresis.

## Results

### Evaluation of specificity and sensitivity of MDV primers by real-time qPCR

**Fig. 1:**
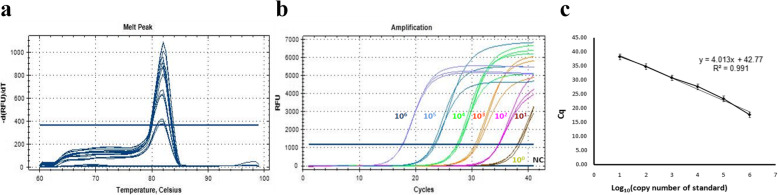
Real-time qPCR analyses of the specificity and sensitivity of primer pair designed for MDV detection. **a** Melting curve analyses; **b** Typical raw fluorescence data from a real-time qPCR assay for the standard MDV plasmid. NC is the negative control. **c** Reproducibility of the real-time qPCR assay. The Cq value is represented as mean ± standard deviation (SD). The standard regression line was generated based on 6 data sets

Real-time qPCR was done to test the specificity and sensitivity of the primer pair designed for MDV detection, and to perform absolute quantification of the copy numbers (Additional file 1: Table S1). There was only a single peak in melting curve analyses following qPCR and sequence analysis of subsequent amplification products confirmed the specific amplification of MDV (Fig. 1a) showing that the primer pair provided highly efficient amplification. As shown in Fig. 1b, the dynamic detection range of the assay spanned 6 logs ranging from 6 to 1 log copies per reaction. Even a MDV template as low as 10^1^ copies/μL could be detected by qPCR. The Cq phase difference between each template concentration gradient was similar, indicating that each template dilution gradient was indeed 10 times the difference (Fig. 1b). There was also a good linear relationship (r value> 0.991) between the Cq values and the log-transformed copy numbers (Fig. 1c). This pair of primers was then employed in PCR and RPA assays for further validation.

### Comparison of the specificity and sensitivity of PCR and RPA using agarose gel electrophoresis

To test the specificity of the primers for use in PCR or RPA under optimal conditions, reactions were performed using DNA of PCV, MDV and TYLCV, and cDNA of TMV, PMMoV, TuMV, CGMMV and RSV. In agarose gel electrophoresis of PCR and RPA products, the expected specific band of about 400 bp was amplified from DNA of MDV-infected plants. No primer dimers were found in either PCR or RPA assays. There were no cross reactions with any of the other viruses, suggesting that the primer pair is specific and suitable for detecting MDV by PCR or RPA (Additional file 2: Figure S1a, S1b).

**Fig. 2:**
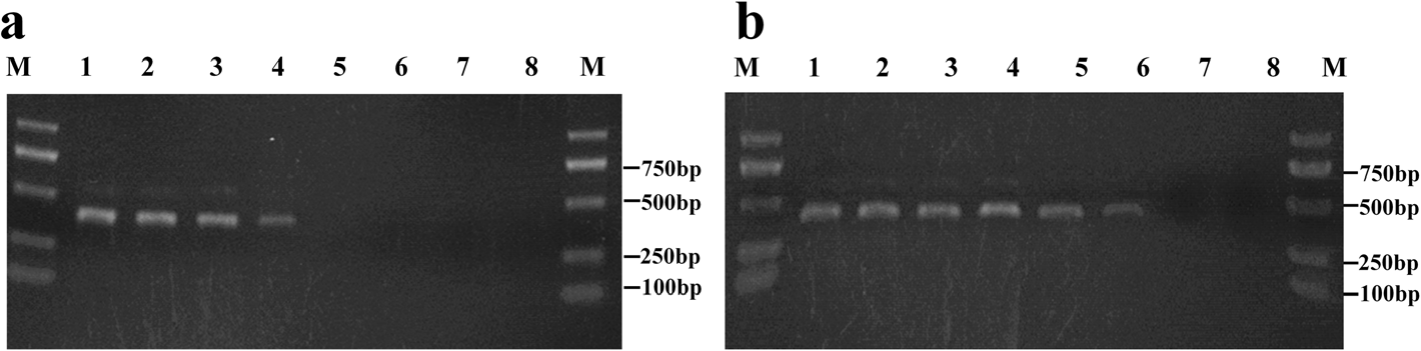
Molecular sensitivity of the MDV PCR and RPA assays by agarose gel electrophoresis. **a** PCR assay; **b** RPA assay. M: Trans2K®Plus DNA Marker; 1: 10^6^ copies; 2: 10^5^copies; 3: 10^4^ copies, 4: 10^3^ copies, 5: 10^2^ copies; 6: 10^1^ copies; 7: 10^0^copy; 8: negative control with crude leaf extracts of healthy cowpea

The sensitivity of the PCR and RPA assays for detecting MDV were compared using a dilution series of the pEASY-T5-MDV plasmid (10^6^ to 10^0^ copies/μL) as templates. From 1.5% agarose gel electrophoresis of the products, PCR had a detection limit of 10^3^ copies/μL (Fig. 2a), while RPA was much more sensitive and gave a strong positive signal from a template of only 10^1^ copies/μL (Fig. 2b).

### Comparison of the sensitivity of PCR and RPA combined with LFS

The sensitivity of PCR or RPA combined with LFS for the detection of MDV was also tested using a dilution series of the MDV plasmid. Both methods were able to detect MDV down to a template of 10^1^ copies/μL (Additional file 3: Figure S2a, S2b). Thus LFS provided a similar sensitivity as agarose gel electrophoresis when combined with RPA but enhanced the sensitivity of PCR detection (Fig. 2 and Fig. S2). Using the same amount of PCR product as substrate, the sensitivity of detection by LFS was at least 100 times higher than that of agarose gel electrophoresis (Additional file 4: Figure S3).

Since the RPA reaction can be completed at 37 °C, the reaction can be done in various conventional instruments. RPA-LFS was able to detect 10^1^ copies of the MDV template with similar efficiency whether the incubation was done in a PCR instrument, a water bath or in an oven (Additional file 3: Figure S2b, S2c, S2d).

### Detection of MDV in crude leaf extracts by PCR-LFS, RPA-LFS and qPCR

**Fig. 3:**
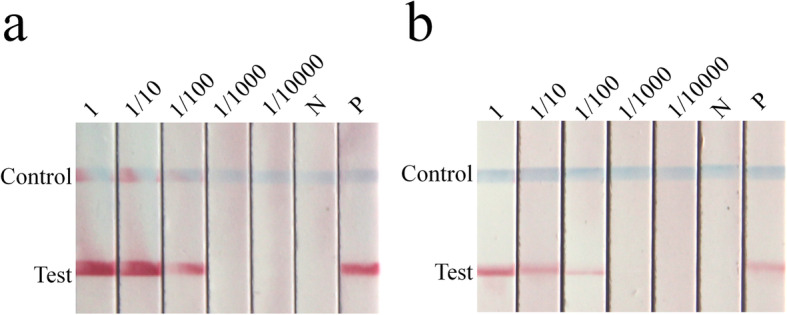
The ability of PCR-LFS and RPA-LFS assays to detect MDV in crude leaf extracts. Crude extract undiluted (1) followed by 1:10, 1:100, 1:1000, 1:10000 dilutions, with healthy cowpea leaf as a negative control (N) and 10^6^ copies of pEASY-T5-MDV plasmid as a positive control (P). **a** Detection of MDV in crude leaf extracts by PCR-LFS; **b** Detection of MDV in crude leaf extracts by RPA-LFS

In order to determine the feasibility of detecting MDV from crude leaf extracts, extracts prepared from leaves of MDV-infected cowpea plants with a DNA concentration of 21.3 ng/μL were used at various dilutions to test the ability of PCR-LFS or RPA-LFS assays to detect the virus. MDV was successfully detected by both methods in undiluted samples and in those diluted 1:10 or 1:100, but there were no positive signals from the uninfected control leaves (Fig. 3). This result suggested that the crude leaf extracts could essentially meet the template requirements for RPA-LFS detection.

**Fig. 4:**
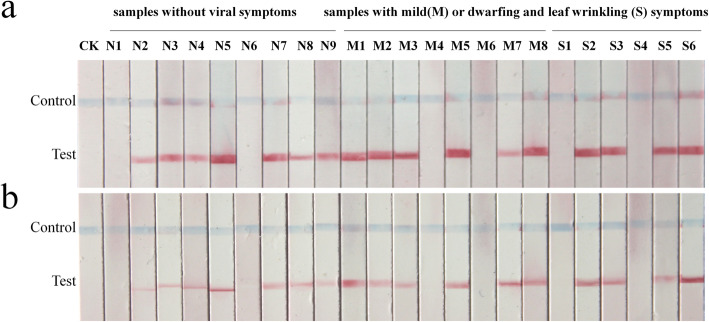
Comparison of PCR-LFS and RPA-LFS assays for detecting MDV in crude leaf extracts of field samples. Nine field samples without viral symptoms, eight field samples with mild viral symptoms, six samples with dwarfing and leaf wrinkling symptoms and healthy cowpea plants without disease symptoms were tested by PCR-LFS and RPA-LFS. PCR-LFS and RPA-LFS assays gave identical results. Of the twenty-three field samples tested, seventeen were positive and six were negative. **a** Detection of MDV in field samples by PCR-LFS; **b** Detection of MDV in field samples by RPA-LFS. N: samples without viral symptoms; M: samples with mild viral symptoms; S: samples with dwarfing and leaf wrinkling symptoms; CK: healthy cowpea plants without disease symptoms, negative control

To test whether the RPA-LFS we had established is suitable for on-site inspection, the method was used with crude leaf extracts prepared from a healthy cowpea plant and from twenty-three randomly selected field samples. Among the field samples, six plants had obvious dwarfing, eight had mild stunting symptoms and nine had no obvious symptoms. These extracts were also tested by PCR-LFS and qPCR for comparative evaluation. The three methods gave consistent results, showing that MDV could be detected in seventeen samples (Fig. 4, Additional file 5:Table S2). Thus, the accuracy of the on-site RPA-LFS method was similar to that of the PCR-LFS and qPCR methods in the laboratory in these crude leaf extracts.

## Discussion

MDV is a DNA virus that mainly infects legume crops, but which more recently has been found to infect tobacco, causing severe symptoms and even the death of the whole plant [6, 7]. The expansion of its host range and the huge economic losses it causes has increased efforts to study the virus, and its rapid detection has become an important issue.

RPA is a novel isothermal DNA amplification and detection technique. It can be conducted at a single constant low temperature of 37 °C, thus avoiding the use of complex thermal cyclers [9]. Reactions can be completed in 5–20 min, depending on the initial copy number of the targeted templates and the size of the amplicon [18]. The method is now widely used for the rapid detection of plant pathogens [19–21]. With the development of comprehensive application technology, RPA-LFS has become a promising technology for plant virus detection due to its speed, sensitivity and convenience [22–25].

In our studies, the specificity of PCR and RPA was verified by real-time qPCR, agarose gel electrophoresis and subsequent sequence analysis, which suggested the primer pair we design was specific for MDV detection both in PCR and RPA. The RPA assays detected by agarose gel electrophoresis or by LFS have the same high sensitivity as that of qPCR, detecting MDV down to a template of 10^1^ copies/μL which was 100 times more sensitive than conventional PCR and agarose gel electrophoresis at 10^3^ copies/μL. Surprisingly, when we further used LFS to detect the PCR and RPA products, the lowest detection thresholds of PCR-LFS and RPA-LFS were both 10^1^ copies, which suggested that LFS was more sensitive than agarose gel electrophoresis for the detection of MDV PCR products. There have been similar reports in the detection of other pathogens [26, 27]. The sensitivity of LFS is higher than that of agarose gel electrophoresis, and so provides a simpler and more convenient way to improve the detection rate of virus. It has been reported that the LAMP method is also 100 fold more sensitive than conventional PCR for detecting MDV [8]. Our RPA assay also gave a positive signal at 10^1^ copies/μL (Fig. 3b) and has the advantages over LAMP that it is not necessary to design two sets of primers or to amplify nucleic acids at 60–65 °C.

The RPA assay developed in this paper could detect MDV in about 30 min at 37 °C with a higher sensitivity than PCR, and needed a shorter time and a lower temperature than the PCR or LAMP techniques. Different incubation methods for RPA all gave similar sensitivity making it a convenient and flexible method. Compared with PCR-LFS and qPCR, RPA-LFS can detect MDV in crude leaf extracts in the field without additional instruments, while achieving the same sensitivity as qPCR and so provides a convenient and fast visual detection method for MDV in field applications. In the early stages when there are no obvious symptoms of virus disease, the on-site detection of MDV with the established RPA-LFS technology can help with the eradication of virus-infected seedlings and the timely control of aphids in the field.

## Conclusions

In conclusion, the MDV RPA assay established in this study can be successfully used for the rapid detection of MDV infected plants. The RPA-LFS assay is a sensitive and specific method for rapid visual detection of MDV. To our knowledge, this is the first report to show the application of RPA and RPA-LFS technology for quick diagnosis of MDV.

## Supplementary information

**Additional file 1 Table S1.** The Cq values in qPCR tests of the sensitivity of MDV detection.

**Additional file 2 Figure S1.** Molecular specificity of the MDV PCR and RPA assays. a) MDV PCR assay; b) MDV RPA assay. M: Trans2K®Plus DNA Marker; 1: pEASY-T5-PCV3; 2: pCB-PMMoV; 3: pCB-CGMMV; 4: cDNA of *N. benthamiana* plants infected by RSV; 5: DNA of TYLCV infected *N. benthamiana* plant; 6: pGR-TuMV-GFP; 7: p35S-30B::GFP; 8: pEASY-T5-MDV.

**Additional file 3 Figure S2.** Sensitivity of the PCR-LFS and RPA-LFS assays for detecting MDV using different incubation methods. a) PCR products incubated in 37 °C PCR instrument; b) RPA products incubated in 37 °C PCR instrument; c) RPA products incubated in 37 °C water bath; d) RPA products incubated in 37 °C oven.

**Additional file 4 Figure S3.** Comparison of sensitivity between gel electrophoresis and LFS detection. M. Trans2K®Plus DNA Marker; 1. PCR products of MDV, the total DNA amount is 76 ng; 2, 3, 4, 5 are the gradient diluted products of 1, the total DNA amounts for agarose gel electrophoresis or LFS detection are 7.6 ng, 760 pg, 76 pg, 7.6 pg, respectively; N. Negative control, ddH_2_O.

**Additional file 5 Table S2.** The Cq values of selected cowpea field plants in MDV detection by qPCR and the corresponding sample information.

## Data Availability

All data generated or analysed during this study are included in this published article and its supplementary information files.

## Abbreviations

ssDNA: Single strand deoxyribonucleic acid
PCR: Polymerase chain reaction
ORF: Open reading frame
RCA: Rolling circle amplification
LAMP: Loop-mediated isothermal amplification
CP: Coat protein
MDV: Milk vetch dwarf virus
RSV: Rice stripe virus
TYLCV: Tomato yellow leaf curl virus
PCV: Porcine circovirus
PMMoV: Pepper mild mottle virus
CGMMV: Cucumber green mottle mosaic virus
TuMV: Turnip Mosaic Virus
TMV: Tobacco mosaic virus
FITC: Fluorescein isothiocyante
qPCR: Quantitative PCR
RPA: Recombinase polymerase amplification
LFS: Lateral flow strip
RPA-LFS: Recombinase polymerase amplification in combination with a simpler lateral flow strip
PCR-LFS: Polymerase chain reaction in combination with a simpler lateral flow strip
