# Supplementary material for: Rapid and visual detection of milk vetch dwarf virus using recombinase polymerase amplification combined with lateral flow strips
Source: Virol J. 2020 Jul 11;17:102. doi: 10.1186/s12985-020-01371-5 (PMC7353715; doi:10.1186/s12985-020-01371-5)
Supplement: Supplementary file 1 — Additional file 1 Table S1. The Cq values in qPCR tests of the sensitivity of MDV detection. [file 12985_2020_1371_MOESM1_ESM.docx]

Table S1. The Cq values in qPCR tests of the sensitivity of MDV detection

| Copies/μL  Cq | 10^6^ | 10^5^ | 10^4^ | 10^3^ | 10^2^ | 10^1^ | 10^0^ | CK |
| --- | --- | --- | --- | --- | --- | --- | --- | --- |
| Repeat 1 | 17.72 | 23.54 | 27.67 | 30.53 | 34.82 | 38.09 | 0.00 | 0.00 |
| Repeat 2 | 17.63 | 23.08 | 27.69 | 30.82 | 34.68 | 38.50 | 0.00 | 0.00 |
| Repeat 3 | 17.62 | 23.33 | 27.42 | 31.10 | 34.74 | 38.15 | 0.00 | 0.00 |
| AVE | 17.66 | 23.32 | 27.59 | 30.82 | 34.75 | 38.25 | 0.00 | 0.00 |
| S D | 0.04 | 0.19 | 0.12 | 0.23 | 0.06 | 0.18 | 0 | 0 |
