# Supplementary figures and images for: Rapid and visual detection of milk vetch dwarf virus using recombinase polymerase amplification combined with lateral flow strips
Source: Virol J. 2020 Jul 11;17:102. doi: 10.1186/s12985-020-01371-5 (PMC7353715; doi:10.1186/s12985-020-01371-5)

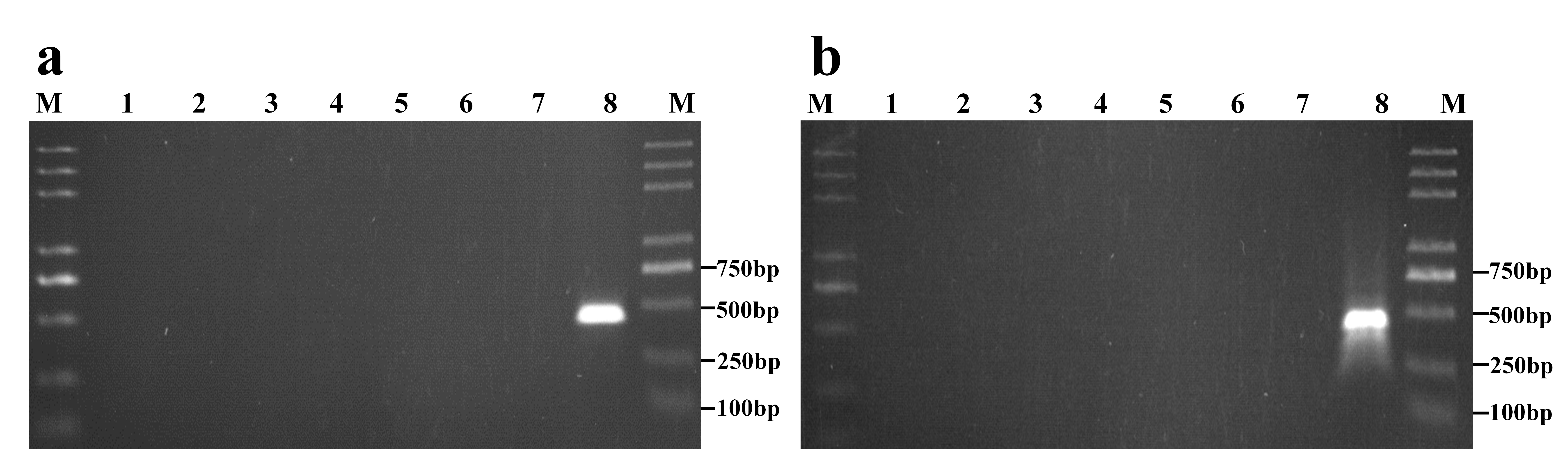

Supplement: Supplementary file 2 — Additional file 2 Figure S1. Molecular specificity of the MDV PCR and RPA assays. a) MDV PCR assay; b) MDV RPA assay. M: Trans2K®Plus DNA Marker; 1: pEASY-T5-PCV3; 2: pCB-PMMoV; 3: pCB-CGMMV; 4: cDNA of N. benthamiana plants infected by RSV; 5: DNA of TYLCV infected N. benthamiana plant; 6: pGR-TuMV-GFP; 7: p35S-30B::GFP; 8: pEASY-T5-MDV. [file 12985_2020_1371_MOESM2_ESM.tif]

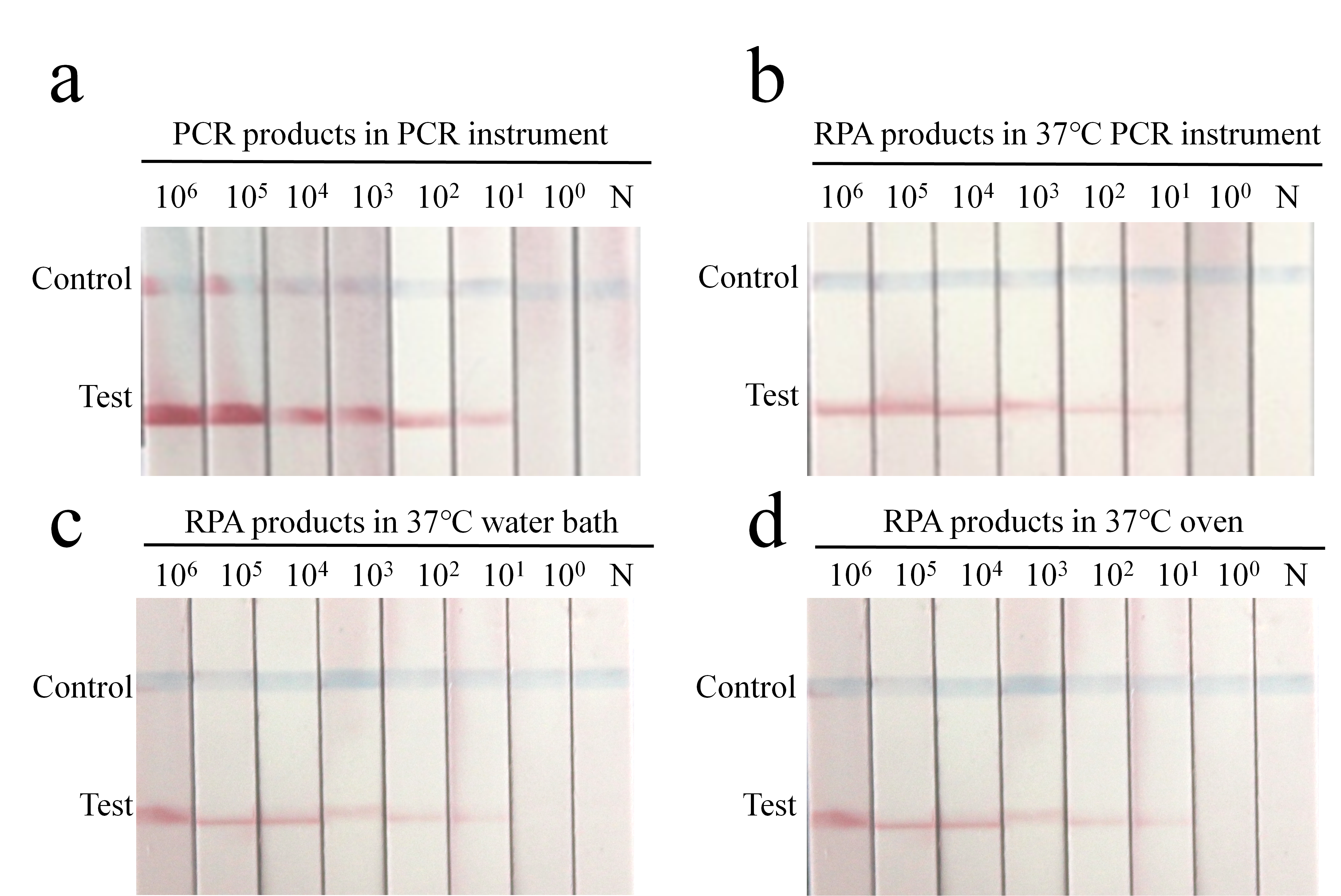

Supplement: Supplementary file 3 — Additional file 3 Figure S2. Sensitivity of the PCR-LFS and RPA-LFS assays for detecting MDV using different incubation methods. a) PCR products incubated in 37 °C PCR instrument; b) RPA products incubated in 37 °C PCR instrument; c) RPA products incubated in 37 °C water bath; d) RPA products incubated in 37 °C oven. [file 12985_2020_1371_MOESM3_ESM.tif]

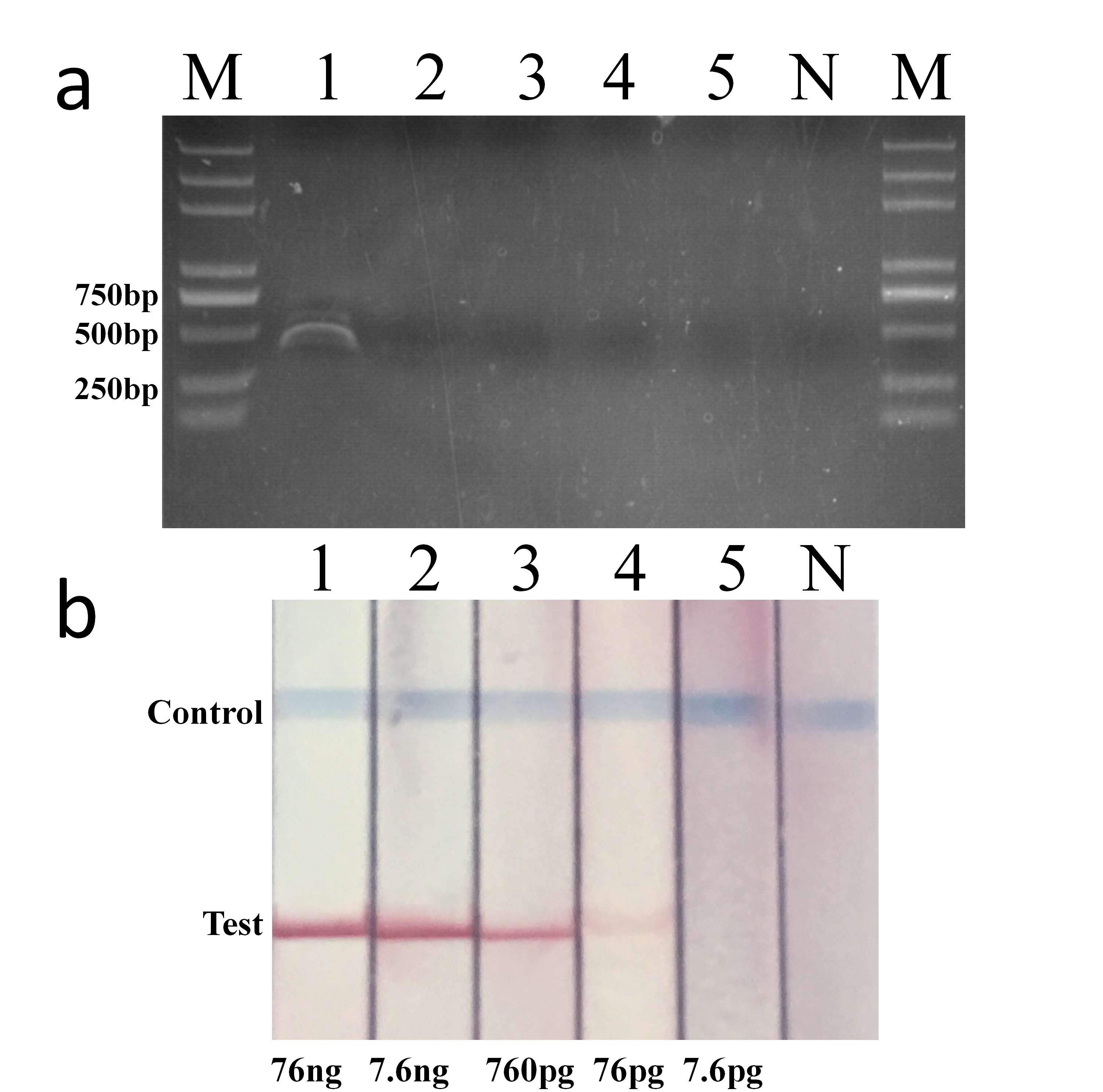

Supplement: Supplementary file 4 — Additional file 4 Figure S3. Comparison of sensitivity between gel electrophoresis and LFS detection. M. Trans2K®Plus DNA Marker; 1. PCR products of MDV, the total DNA amount is 76 ng; 2, 3, 4, 5 are the gradient diluted products of 1, the total DNA amounts for agarose gel electrophoresis or LFS detection are 7.6 ng, 760 pg, 76 pg, 7.6 pg, respectively; N. Negative control, ddH2O. [file 12985_2020_1371_MOESM4_ESM.tif]
