# Supplementary material for: Rapid and visual detection of milk vetch dwarf virus using recombinase polymerase amplification combined with lateral flow strips
Source: Virol J. 2020 Jul 11;17:102. doi: 10.1186/s12985-020-01371-5 (PMC7353715; doi:10.1186/s12985-020-01371-5)
Supplement: Supplementary file 5 — Additional file 5 Table S2. The Cq values of selected cowpea field plants in MDV detection by qPCR and the corresponding sample information. [file 12985_2020_1371_MOESM5_ESM.doc]

Table S2 The Cq values of selected cowpea field plants in MDV detection by qPCR and the corresponding sample information.

| **Sample code** |  | **Cq** |  | **Average value** | **Symptoms** | **DNA concentration**  **( ng/μL)** | **+/-** |
| --- | --- | --- | --- | --- | --- | --- | --- |
|  | Repeat 1 | Repeat 2 | Repeat 3 |  |  |  |  |
| **CK** | 0.00 | 0.00 | 0.00 | 0.00 | N | 2.95 | - |
| **N1** | 0.00 | 0.00 | 0.00 | 0.00 | N | 3.56 | - |
| **N2** | 36.35 | 35.64 | 36.12 | 36.04 | N | 5.9 | + |
| **N3** | 33.20 | 33.21 | 33.03 | 33.15 | N | 16.7 | + |
| **N4** | 35.15 | 35.14 | 34.96 | 35.08 | N | 17.2 | + |
| **N5** | 17.02 | 17.20 | 17.48 | 17.23 | N | 19.1 | + |
| **N6** | 0.00 | 0.00 | 0.00 | 0.00 | N | 5 | - |
| **N7** | 29.07 | 29.00 | 28.74 | 28.94 | N | 11.2 | + |
| **N8** | 34.88 | 35.18 | 35.57 | 35.21 | N | 6.1 | + |
| **N9** | 33.28 | 32.65 | 33.41 | 33.11 | N | 14.2 | + |
| **M1** | 20.95 | 20.91 | 21.04 | 20.97 | M | 24.2 | + |
| **M2** | 19.59 | 19.03 | 19.07 | 19.23 | M | 20.8 | + |
| **M3** | 22.69 | 23.14 | 23.39 | 23.07 | M | 23.7 | + |
| **M4** | 0.00 | 0.00 | 0.00 | 0.00 | M | 3.43 | - |
| **M5** | 19.85 | 19.04 | 19.20 | 19.36 | M | 21.3 | + |
| **M6** | 0.00 | 0.00 | 0.00 | 0.00 | M | 5.1 | - |
| **M7** | 40.15 | 40.91 | 37.61 | 39.56 | M | 14.1 | + |
| **M8** | 25.71 | 25.56 | 26.00 | 25.76 | M | 13.2 | + |
| **S1** | 0.00 | 0.00 | 0.00 | 0.00 | S | 3.56 | - |
| **S2** | 17.04 | 17.22 | 17.13 | 17.13 | S | 18.2 | + |
| **S3** | 17.70 | 17.71 | 17.58 | 17.66 | S | 21.3 | + |
| **S4** | 0.00 | 0.00 | 0.00 | 0.00 | S | 16.2 | - |
| **S5** | 26.18 | 26.06 | 26.19 | 26.14 | S | 10.9 | + |
| **S6** | 19.24 | 19.29 | 19.41 | 19.31 | S | 18.1 | + |

N: samples without viral symptoms; M: samples with mild viral symptoms; S: samples with dwarfing and leaf wrinkling symptoms; CK: healthy cowpea plants without disease symptoms, negative control.

+: indicated MDV could be detected from crude leaf extracts by qPCR; and -: indicated MDV could not be detected from crude leaf extracts by qPCR.
